# Duck Eggshell Crack Detection by Nondestructive Sonic Measurement and Analysis

**DOI:** 10.3390/s21217299

**Published:** 2021-11-02

**Authors:** Chia-Chun Lai, Cheng-Han Li, Ko-Jung Huang, Ching-Wei Cheng

**Affiliations:** 1 Department of Bio-Industrial Mechatronics Engineering, National Chung Hsing University, Taichung 402, Taiwan; s39911050@gmail.com (C.-C.L.); cghan@smail.nchu.edu.tw (C.-H.L.); g8925008@gmail.com (K.-J.H.); 2 Department of Computer Science and Information Engineering, National Taichung University of Science and Technology, Taichung 404, Taiwan

**Keywords:** nondestructive testing, sound detection, calibration curve, duck eggs, cracked eggshell

## Abstract

Duck eggs are a good source of essential nutrients for the human body. However, transportation, processing, and handling can easily cause cracks in the eggshells. These cracks can lead to microbial contamination, reducing the shelf life and compromising food safety. In this study, a method for the nondestructive testing of cracks in duck eggshells was developed. First, the acoustic emission signals of intact and cracked eggshells were measured, and the most significant frequency features were selected to establish a calibration curve for cracked eggshells. Logistic regression using the frequency features was then adopted to predict intact and cracked eggshells. Then, we establish a set of optimal regression models and used independent samples for verification. The overall accuracy rates of the calibration and prediction models using five frequencies of bandwidth (1500, 5000, 6000, 8500, and 10,000 Hz) were 89.7% and 87.6%, respectively. Sound measurement enables a simple and quantitative method for duck egg crack detection and classification. This nondestructive and cost-effective method can be used for duck egg quality screening and can be integrated into duck egg processing machinery.

## 1. Introduction

Eggs are a rich source of nutrients [1] and are easily absorbed and digested by the human body [2]. They provide amino acids, vitamins, minerals, and high-quality protein required by the human body [3]. Eggs are often consumed at breakfast and are a staple ingredient in baked goods, sweets, sauces, and savory dishes [4].

For human consumption, eggs from chickens and ducks are commonly used. According to statistics from the Duck Breeding Association of the Republic of China, Taiwan’s annual production of duck eggs is approximately 460 million, with a total annual output value of approximately 56 million US dollars [5]. Traditionally, duck eggs can be boiled and eaten directly; they can also be used as raw materials for processing. However, during transportation, the small cracks occurring in eggshells can easily cause leakage and contamination, leading to a reduction in the shelf life of fresh eggs and food products [6]. Therefore, eggs must be carefully screened before entering the market [7]. Duck eggs are the main raw material used for preserved and salted eggs. Cracks on the eggshell will affect the quality and success rate of processing. The manufacturing process of preserved eggs involves interactions between eggs and alkaline substances. If the eggshell is broken, this interaction will be affected, and the production of the preserved eggs will fail. Therefore, the production process for preserved eggs has considerably strict requirements pertaining to the integrity of eggshells. Currently, processing factories typically use light inspection or manual collision methods to assess whether there are cracks in the eggshell. The light inspection method employs light to determine the intact state of the eggshell by manual or machine vision and image analysis. Typically, workers distinguish eggshell cracks based on the sound produced by the slight collision of two or more eggs, as the sound of an intact egg is sharp, whereas the sound of a cracked egg is dull [8]. The types of cracked eggs include minor stripe-marked eggs (MEs), severe stripe-marked eggs (SEs), Cracked eggs (CEs), and broken eggs (BEs). A description is given in Table 1.

Nondestructive testing methods that have been used to detect the integrity of eggshells, such as optical image recognition [10,11] and sound detection [12], are the most widely used techniques. Propagated sound waves interact with the target products, such as duck eggs, producing acoustic emission spectra with characteristic features owing to the transmission, scattering, absorption, and reflection. Information from these detected acoustic signals, such as the propagation speed, acoustic impedance, natural frequency, and other parameters, can be extracted and used to determine the structural properties of the target product [13,14]. Therefore, sound detection is often used for brittle or crispy foods [15]. The quality and intensity of the sound signal produced by a damaged eggshell largely depend on its internal structure (including internal impurities) and the thickness and texture of the eggshell [16,17]. However, previous research has mainly focused on chicken eggs [18,19]. The eggshell structure and thickness of chicken eggs and duck eggs differ [20], thus affecting the response frequency of the sound signals.

Studies have demonstrated that sound measurement and analysis for eggshell crack detection is feasible, and various techniques have been used to extract and analyze the acoustic response of eggshells to classify eggs and detect cracks [18,21]. Most of these methods employ automated learning algorithms, which can be complicated and require a large amount of computing resources; thus, they are not practical or cost-effective, especially when integrated with processing equipment and machinery.

In this study, a method was developed and evaluated for the nondestructive testing and crack detection of duck eggshells. A calibration curve for eggshell crack detection was derived by measuring the sound signals from intact and cracked eggs and selecting the most significant frequency features. This nondestructive crack detection technology can be useful for the automated screening of the quality of duck eggs and can also be integrated with duck-egg-processing machinery.

## 2. Materials and Methods

### 2.1. Sample Selection

The eggs used in this study were fresh eggs laid by Brown Tsaiya ducks (scientific name: *Anas platyrhynchos* var. *domestica*) that were 25–28 weeks old and were purchased from duck farms. The egg shape index is between 0.69 and 0.74. The duck eggs were manually screened, transported to the laboratory and detected under constant temperature and humidity conditions (23–26 °C, 55% RH). Initially, 60 duck eggs were sampled, and sound signal data were collected and analyzed to establish a calibration curve. Manual inspection of this batch identified 30 duck eggs with intact eggshells and 30 duck eggs with cracked eggshells. The eggshell cracks were formed during the transportation or production process. The types of eggshell cracks include minor stripe-marked eggs, severe stripe-marked eggs and cracked eggs. A second batch, comprising 66 duck eggs, was used to validate the calibration curve. Manual inspection of the second batch revealed 34 duck eggs with intact eggshells and 32 duck eggs with cracked eggshells.

### 2.2. Measurement Setup

**Figure 1:**
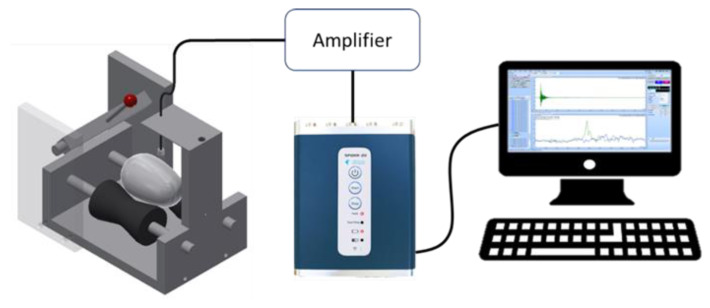
Schematic of measurement setup.

**Figure 2:**
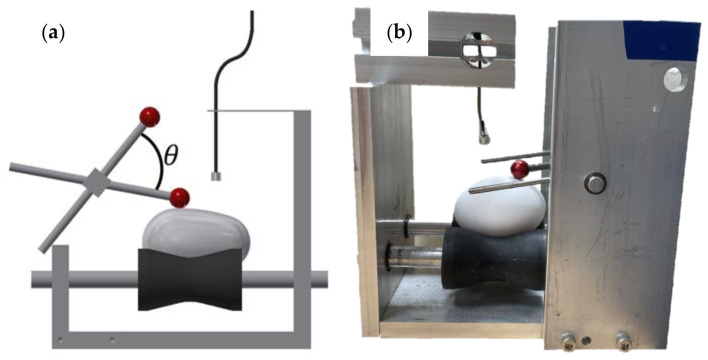
Percussion rod action device (**a**) schematic diagram (**b**) actual device.

The measurement apparatus consisted of an egg wheelset, a percussion rod, and an electret microphone (O650-40C1-L00-00-D, Nessie Industrial Co., Taiwan, Sensitivity: −40 ± 3 dB), as shown in Figure 1. The microphone was installed above the egg and was connected to a frequency spectrum analyzer (SPIDER-20E, Magicdot Co., Ltd., Taiwan, Sampling Rate: 0.48 Hz to 102.4 kHz, with 54 stages; Maximum Useful Bandwidth: 46.08 kHz) via a signal amplifier (2017FFTV1, Broad Products Design Co., Ltd., Taiwan). A desktop computer was used to record and process the acoustic signal data. The percussion rod was 100 mm long and had a plastic sphere with a diameter of 12 mm on one end that was used to strike the eggshell. To standardize the percussive force, the percussion rod was withdrawn to the same height for each percussive strike; the included angle (*θ*) was approximately 60°. The percussion rod was allowed to fall naturally and the plastic ball hit the egg (Figure 2) to ignore the impact of the percussion force on the research results. According to Sun et al. [22], a duck eggshell can withstand a percussive force of 5 kg (49 N). The percussion instrument used in this study (Figure 2) exerts a percussive force of 38 N. Therefore, it would not cause the eggshell to crack. According to Cheng et al. [12], the best position for receiving the sound is above the equator of the egg; hence, the electret microphone was placed 5 mm above the equator.

### 2.3. Sound Signal Sampling

Each duck egg was hit five times, and the acoustic signal was sampled. After each strike, the signal from the microphone was amplified, converted, and sent to the computer. The signals were recorded and analyzed using the proprietary software of the frequency spectrum analyzer manufacturer. The time-domain signal acquired after the tapping was drawn into a time-domain diagram, and this signal was converted into a frequency-domain signal by Fast Fourier Transform (FFT). The frequency bandwidth was 0–10, 240 Hz, the sampling rate was 20,480, the number of sampling points was 2,048, and the sampling time was 100 ms. The data were subsequently imported into statistical analysis software.

### 2.4. Logistic Regression

Classification and modeling are based on predictions that are dependent on the chosen variables and the collected data [23]. A regression analysis is often used to analyze the relationship between independent and dependent variables [24,25]. Linear regression is mainly used for regression problems, while logistic regression is usually used for classification problems. For dependent variables, the dependent variable in multiple linear regression (MLR) is continuous, while the dependent variable in logistic regression is discrete. There are also differences in the assumptions made for each type of regression. In MLR, the variable data are assumed to follow a normal distribution; however, in logistic regression, the cumulative probabilities of dependent variable outcomes are assumed to exhibit S-shaped distributions (also known as logistic distributions). Logistic regression is a statistical method for modeling the probability of a binary dependent variable, and it is utilized in supervised learning methods [26]. It can also be used to consider the influence of several factors on the probability of any event [27,28,29], and this is used in acoustics [30,31,32].

In this study, the state of the eggshells is divided into two types: intact eggshells, and cracked eggshells. This is a classification problem. In this model, whether the eggshell has cracks was regarded as the dependent variable. The dependent variable is binary [33], where “0” implies the presence of cracks and “1” indicates no cracks. Therefore, logistic regression technology is used to analyze whether the duck eggshell has cracks. SPSS (version 20.0) was used to integrate the characteristic frequencies of the selected non-cracked eggshells into the logistic regression model. The probability of intact eggs is

(1) $$p=\frac{1}{1+e^{-z}}$$

where *p* is the probability of occurrence of an intact egg, and *z* is the linear regression function:

(2) $$z=b_{1}x_{1}+b_{2}x_{2}\ldots b_{n}x_{n}+c$$

where *b_n_* is the coefficient of the logistic regression model, *x_n_* is the selected frequency feature, and *c* is a constant, that is, the vertical-axis intercept of the logistic regression model. If the coefficient of the model is positive and has a large magnitude, the characteristic frequency has a significant impact on the probability of occurrence of an intact egg. Conversely, a negative coefficient with a large magnitude indicates that the probability of the cracked eggs increased. The mathematical expression for the probability of a cracked egg is

(3) $$1-p=1-\frac{1}{1+e^{-z}}=\frac{1+e^{-z}-1}{1+e^{-z}}=\frac{e^{-z}}{1+e^{-z}}$$

### 2.5. Receiver Operating Characteristic (ROC) Curve

Machine learning often uses binary classification methods to represent classification or prediction results in a confusion matrix. The confusion matrix can easily judge whether there are misjudgments and misclassifications of different kinds of objects. The confusion matrix can calculate the sensitivity and specificity of the classifier and plot it into the receiver operating characteristic curve [5]. The ROC curve shows the performance of a binary classifier system for a specific classification or discrimination threshold. In this study, ROC curves were created using two parameters, sensitivity and 1-specificity, which are represented by the vertical and horizontal axes, respectively [24]. Confusion matrix and ROC curves are widely used in medical fields [34,35,36,37]. The eggshells were divided into two types: intact eggshells or cracked eggshells. The measured prediction results were classified according to the actual condition of the eggshells, as determined via manual inspection. If an eggshell was intact, it was classified as true positive (*TP*) if the prediction result was an intact eggshell. If the predicted result was a cracked eggshell but the eggshell was actually intact, it was classified as a false negative (*FN*). In the case of cracked eggshells, if the predicted result was a cracked eggshell, it was classified as true negative (*TN*), and if the predicted result was an intact eggshell, it was classified as a false positive (*FP*). The confusion matrix is shown in Table 1. The accuracy was calculated as the ratio of the number of correct predictions to the total number of predictions. The equations for sensitivity, specificity, and accuracy are as follows:

(4) $$\mathrm{Sensitivity}=\frac{TP}{TP+FN}$$

(5) $$\mathrm{Specificity}=\frac{TN}{TN+FP}$$

(6) $$\mathrm{Accuracy}=\frac{TP+TN}{TP+TN+FP+FN}$$

In the ROC method, model validation is performed using the area under the curve (AUC). The higher the AUC value, the better the performance of the model [35]. The AUC is typically in the range of 0.5–1 and is divided into five standards: weak (0.5–0.6), moderate (0.6–0.7), good (0.7–0.8), very good (0.8–0.9), and excellent (0.9–1) [38,39].

## 3. Results and Discussion

### 3.1. Signal Analysis

**Figure 3:**
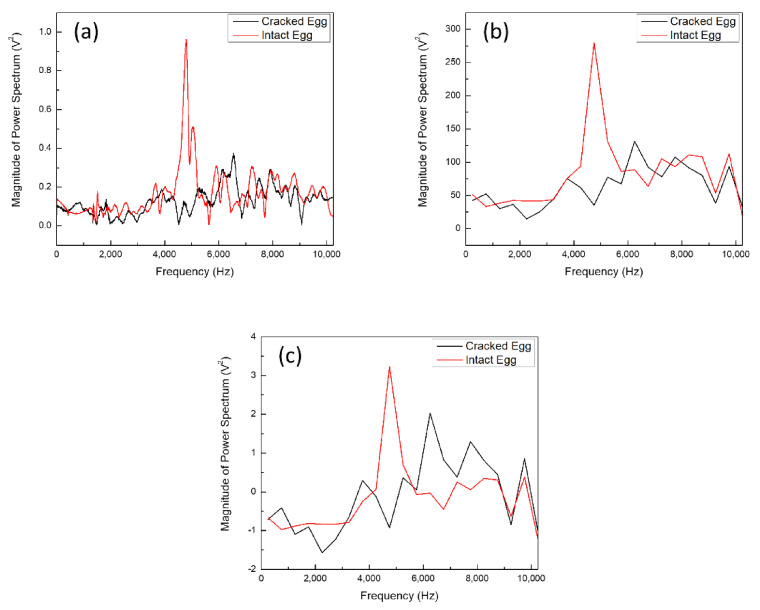
(**a**)Typical (**b**) Integrate (**c**) Normalized frequency-domain spectra of intact eggs (red line) and cracked eggs (black line).

The acquired signal can be analyzed in the time domain or the frequency domain. The signal in the time domain is composed of one or more sine waves of frequency, amplitude and phase superimposed, through Fourier transform into the corresponding frequency domain signal, to obtain the energy value of the signal at a specific frequency; hence, a Fast Fourier Transform (FFT) is used to convert time-domain data into frequency-domain data. For the frequency domain, the typical spectra of the intact and cracked eggs are shown in Figure 3a. As can be observed, it is easy to spectroscopically differentiate between intact and cracked eggs. The intact egg has an obvious peak in the frequency bandwidth of 4000–6000 Hz, while the cracked egg does not. However, the frequency is easily shifted by factors such as eggshell quality, composition and thickness. In order to make the calibration curve universal and to eliminate the influence of environmental factors, the obtained data are integrated with a unit bandwidth of 500 Hz to reduce the impact of displacement (Figure 3b), and eliminate environmental factors through normalization. The final data are shown in Figure 3c. The processed data are no different from the original data. Therefore, these data were used to establish a calibration curve for eggshell cracks. Therefore, the normalized data were used to establish a calibration curve for eggshell cracks.

### 3.2. Generating Eggshell Crack Calibration Curve

In this experiment, 30 duck eggs with intact eggshells and 30 cracked eggs were sampled. Each duck egg was tapped five times; thus, 60 × 5, or 300 datapoints were obtained. The data were imported into the statistical analysis software package SPSS for logistic regression analysis. Forward stepwise regression analysis was used to select the frequency features. Stepwise regression analysis identifies the factor that predicts the dependent variable most among the many independent variables. The results identify the key independent variables that affect the dependent variables and prioritize select the independent variables that are more closely related to the dependent variables [40]. The results of the analysis are presented in Table 2.

**Figure 4:**
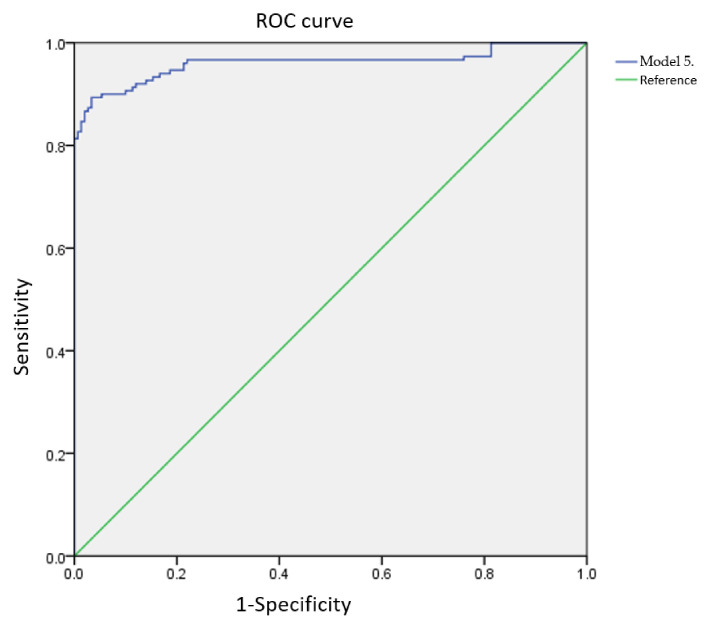
Model 5 receiver operating characteristic (ROC) curve for the calibration group.

The calibration equations established using the calibration group were used to predict the sample outcomes of the validation group. Using a single-frequency feature bandwidth (6000 Hz), the Nagelkerke *R*^2^ coefficient of determination was 0.417. When four frequency features bandwidths (Model 5; 1500, 5000, 6000, 8500, and 10,000 Hz) were used, the Nagelkerke *R*^2^ coefficient was 0.946, indicating that the model is highly predictive of eggshell cracks. The model fit and parameter significance for Model 5 are summarized in Table 3. The ROC curve is shown in Figure 4. The AUC of the calibration group for Model 5 was 0.96.

**Table 2:** Accuracy of four models of eggshell crack detection by the calibration set.

Model	Selected Frequencies Bandwidth (Hz)	Nagelkerke *R*^2^	Intact Egg			Cracked Eggs			Overall Accuracy (%)
			True (units)	False (units)	Accuracy (%)	True (units)	False (units)	Accuracy(%)	
1	6000	0.379	117	33	78	100	50	66.7	72.3
2	5000, 6000	0.602	128	22	85.3	124	26	82.7	84
3	1500, 5000, 6000	0.682	133	17	88.7	133	17	88.7	88.7
4	1500, 5000, 6000, 10,000	0.727	140	10	93.3	137	13	91.3	92.3
5	1500, 5000, 6000, 8500, 10,000	0.776	137	13	91.3	132	18	88	89.7

**Table 3:** Fit of Model 5 eggshell crack calibration curve for the prediction of eggshell cracks.

i (1-n)	Frequency (Hz)	b_i_	Std. Error	Wald Test	Variance Inflation Factor (VIF)
1	1500	−2.461	0.664	13.721	1.076
2	5000	1.547	0.253	37.305	1.121
3	6000	−2.246	0.326	47.439	1.074
4	8500	1.853	0.420	19.432	1.212
5	10,000	−2.112	0.398	28.172	1.277
c	–	−5.690	0.898	40.116 ***	–
Overall model fit		$\chi^{2}$ = 261.753 *** Hosmer–Lemeshow = 136.445 ^n.s.^			

Note. *** *p* < 0.001; n.s. > 0.05.

Table 2 shows that the use of five frequency bandwidths (1500, 5000, 6000, 8500, and 10,000 Hz) for the regression model allows for eggshell cracks to be predicted with high accuracy. The calibration curves of the five characteristic frequency bandwidths (1500, 5000, 6000, 8500, and 10,000 Hz (Figure 4)) were combined to generate ROC curves for intact and cracked eggs. The intact egg AUC was 0.96. Hence, the logit equation was established as

(7) $$f\left(x\right)=\left(-5.69\right)-2.246\times f\left(6000\right)+1.547\times f\left(5000\right)-2.461\times f\left(1500\right)-2.112\times f\left(10,000\right)+1.853\times f\left(8500\right)$$

**Figure 5:**
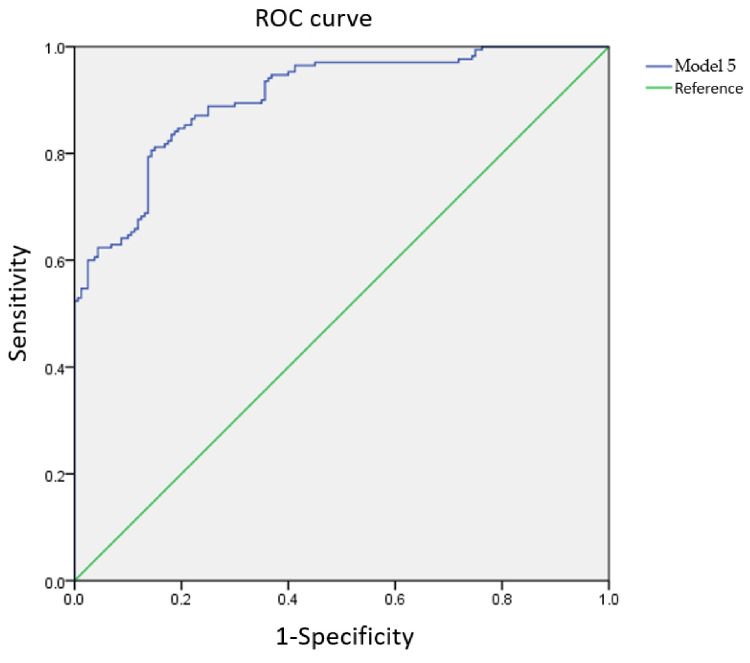
Model 5 ROC curve for the validation group.

To test the accuracy of the calibration curve, 34 duck eggs with intact eggshells and 32 cracked duck eggs were used for validation. Each duck egg was tapped by the percussion rod five times, and a total of 66 × 5, or 330 datapoints, were obtained for the logistic regression analysis. Model 5, with five input frequencies, was used. The Nagelkerke *R*^2^ value of the validation group was 0.729, and the duck eggs were manually inspected for cracks to establish the corresponding confusion matrix, as shown in Table 4. The ROC curve for the validation group is shown in Figure 5. The calculated AUC value was 0.905.

**Table 4:** Model 5 confusion matrix for the validation group.

Prediction Frequency Bandwidth (Hz)		1500, 5000, 6000, 8500, 10,000		
State		Predicted		Accuracy
		Intact	Crack	
Actual	Intact	151	19	88.8%
	Crack	22	138	86.3%
Overall accuracy				87.6%
Nagelkerke R^2^				0.729

Manual detection involves distinguishing the sound of eggshell collisions aurally. In an actual processing plant, the noise of the environment affects the workers’ ability to listen to the sound of eggshell collisions. Furthermore, workers need to test thousands of duck eggs every day. Long-term manual testing will reduce the accuracy of detection and even cause occupational injuries. Therefore, a machine learning method is proposed with a high detection accuracy and ease of use. This method uses sound resonance frequency analysis to detect cracks in duck eggshells. Table 3 shows that, in all four models, a frequency of 5000 Hz is present, indicating that this frequency is the dominant frequency of an intact eggshell. This result is consistent with the result of Cheng et al. [12]. They analyzed duck eggshells with the SVM training method and found that the characteristic frequency of a complete duck eggshell was between 4130 Hz and 5500 Hz, and the accuracy rate was 98%. In addition, Botta et al. [41] used image capture and collocation with CNN training to detect eggshell cracks with an accuracy rate of 99.5%. This demonstrates that this method can be applied to the detection of eggshell cracks.

## 4. Conclusions

In this study, the acoustic emissions of duck eggshells were measured and logistic regression analysis was performed. The following conclusions can be drawn:

First, the five significant frequency features (1500, 5000, 6000, 8500, and 10,000 Hz) formed a highly accurate predictive model for classifying intact and cracked duck eggshells. The Nagelkerke *R*^2^ value and AUC of the calibration group were 0.776 and 0.960, whereas those of the validation group sample were 0.729 and 0.876, respectively. These results indicate that the model is highly correlated with the presence/absence of eggshell cracks and can predict whether the eggshell is intact or cracked.

This study used acoustic signal detection and analysis to provide an objective and simple method for detecting cracks in duck eggs. This method can determine the characteristic frequencies of intact and cracked duck eggs. In the future, when designing automatic duck eggshell crack detection equipment, the frequency band can be used to reduce the misjudgments caused by different impact forces and environmental factors. This nondestructive eggshell crack detection method is highly accurate and economical, and can be used for the automated and large-scale quality screening of duck eggs.

## Figures and Tables

**Table 1:** Cracked egg types and description. [9].

Category	Description
Minor stripe-marked egg (ME)	An egg that has a gray stripe-mark (<2 cm), but no damage has occurred to the eggshell membrane and no egg components have leaked from the egg.
Severe stripe-marked egg (SE)	An egg that has a single gray stripe mark or for which the sum of the lengths of individual stripe marks is more than 2 cm, but no damage has occurred to the eggshell membrane and no egg components have leaked from the egg.
Cracked egg (CE)	An egg that has at least one visible hair-like microcrack on the eggshell, but no damage has occurred to the eggshell membrane and no egg components have leaked from the egg.
Broken egg (BE)	An egg that has at least one complete eggshell crack or hole, where the eggshell and shell membrane have broken and egg components have leaked from the egg.

## Data Availability

Not applicable.
